# Counterfactual Reasoning for Regretted Situations Involving Controllable Versus Uncontrollable Events: The Modulating Role of Contingent Self-Esteem

**DOI:** 10.5709/acp-0168-4

**Published:** 2015-03-31

**Authors:** Meredith R. Wilkinson, Linden J. Ball, David Alford

**Affiliations:** 1 Division of Psychology, De Montfort University, Leicester, UK; 2 School of Psychology, University of Central Lancashire, Preston, UK; 3 School of Psychology, University of East London, London, UK

**Keywords:** contingent self-esteem, regret, counterfactual reasoning, controllable events, uncontrollable events

## Abstract

We report a study that examined the modulating impact of *contingent* self-esteem on regret intensity for regretted outcomes associated with controllable versus uncontrollable events. The Contingent Self-Esteem Scale (e.g., Kernis & Goldman, 2006) was used to assess the extent to which a person’s sense of self-worth is based on self and others’ expectations. We found that there was an influence of self-esteem contingency for controllable but not for uncontrollable regret types. For controllable regret types individuals with a high contingent (i.e., unstable) self-esteem reported greater regret intensity than those with a low contingent (i.e., stable) self-esteem. We interpret this finding as reflecting a functional and adaptive role of high contingent self-esteem in terms of mobilizing the application of counterfactual reasoning and planning mechanisms that can enable personal expectations to be achieved in the future.

## Introduction

Counterfactual reasoning is a pervasive cognitive activity that occurs whenever we imagine how outcomes could have turned out differently, either for the better or for the worse. When counterfactual reasoning arises in the wake of a negative outcome it typically gives rise to feelings of regret (sometimes of a profound nature), since people envisage how things could have worked out better than they did. The link between counterfactual reasoning and regret has engendered considerable research interest over the past 30 years or so, with studies having identified a number of important phenomena. One example is the so-called “action effect” (see Kahneman & Tversky, 1982), which is the tendency to regret *action* more in the short term but to regret *inaction* more in the long term (e.g., Gilovich & Medvec, 1994; Kahneman & Tversky, 1982; Morrison & Roese, 2011). Another example is the “temporal order effect”, whereby people are more likely to reason about “undoing” the final event in a sequence of events that led to a negative outcome, rather than undoing any preceding events (e.g., Byrne, Segura, Culhane, Tasso, & Berrocal, 2000).

More recent research has started to examine the connections between person variables and counterfactual reasoning, including the association between self-esteem and perceived regret intensity (e.g., Feeney, Gardiner, Johnston, Jones, & McEvoy, 2005; Libby, Valenti, Pfent, & Eibach, 2011) and the links between depressive symptomology and counterfactual thinking about negative personal events (e.g., Markman & Miller, 2006). The present paper examines a pattern of associations that has not previously been investigated—that is, associations between self-esteem and counterfactual reasoning for regretted incidents in which some of the events leading to the negative outcome are either under the protagonists’ control or are outside of their control. Before elaborating on the predictions relating to this study we first overview relevant aspects of the literature on self-esteem, counterfactual reasoning, and regret.

## Self-Esteem, Counterfactual Reasoning, and Regret

Self-esteem is the sense of self-worth that is possessed by a person and it has been shown to influence how people reason about incidents that give rise to feelings of regret. Research examining the association between self-esteem and counterfactual reasoning has often used the Rosenberg Self-Esteem Scale (RSES; Rosenberg, 1965), which is a 10-item measure of state self-esteem based on responses to questions such as “At times I think I am no good at all” and “I wish I could have more respect for myself”. For example, Feeney et al. (2005), adopting the RSES, asked individuals to recall regrets, either from the recent past or, in a second study, from across their entire life. Feeney et al. found that individuals with high self-esteem recalled more regrets associated with inaction than action, whilst low self-esteem individuals showed an even spread of action and inaction regrets. Feeney et al. concluded that these effects arise because high self-esteem people seek to “self-enhance” by distancing themselves from having responsibility for bad outcomes. Recalling inaction regrets therefore makes sense in terms of this self-enhancement motivation since inaction is seen as being less causal of a negative outcome than is action (see Spranca, Minsk, & Baron, 1991).

Feeney et al.’s (2005) proposal concerning the self-enhancing nature of high self-esteem resonates with findings from a previous study by Roese and Olson (1993), who asked participants to imagine themselves in a situation where they were with another actor and the events resulted in a negative outcome (e.g., a bad grade on a group project). Roese and Olson obtained a measure of self-esteem using the Texas Social Behavior Inventory (TSBI; Helmreich & Stapp, 1974), which examines “social” self-esteem, such as perceived attractiveness and ability in social environments (i.e., the TBSI measures a specific rather than a general form of self-esteem, as is the case with the RSES). Roese and Olson found that high self-esteem participants were less likely than low self-esteem participants to change an aspect of their own behaviour in order to undo a negative outcome. Thus, low self-esteem individuals generated “self-referent” counterfactuals such as “If only I had worked harder then I would have received a better mark”, whereas high self-esteem individuals generated “other-referent” counterfactuals such as “If only the other members of my group had worked harder then I would have received a better mark”. Again, then, it appears that high self-esteem people engage in self-enhancing attributions when assessing the causal determinants of a negative outcome that they were associated with. When viewed together, the findings of Feeney et al. (2005) and of Roese and Olson indicate that the self-enhancement effects associated with individuals with high self-esteem generalize across both global and specific self-esteem measures.

Although extant research has provided valuable insights into the relation between self-esteem and counterfactual reasoning with regret-oriented scenarios, it is questionable whether the use of the RSES and TBSI have provided sufficient clarity as to the associations that might exist. Part of the problem here concerns the fact that self-esteem is, in fact, a highly heterogeneous and nuanced construct (e.g., Zeigler-Hill, Fulton, & McLemore, 2011). It has, for example, been suggested that self-esteem can be *stable* or *unstable*, depending upon the extent to which a person’s self-esteem fluctuates over time and context (Kernis, 2005; Kernis & Waschull, 1995).

Very closely related to the concept of stability is the notion of *contingency*, which is the extent to which a person’s sense of self-worth is dependent upon living up to their own and others’ expectations (e.g., Deci & Ryan, 1995; Kernis, 2005; see also Kernis, 2003, for evidence that self-esteem *contingency* and stability are highly correlated). “True” self-esteem (Deci & Ryan, 1995) is therefore viewed as being stable and non-contingent and refers to feelings of self-worth that are well anchored and secure, such that they do not depend on the attainment of particular outcomes and are not in need of constant validation. Viewing self-esteem in this manner, rather than focusing on a specific factor such as social self-esteem, which the TSBI does, enables us to have a more detailed grasp of the nuances and complexities underlying the relationship between self-esteem and regret.

As we explain in our method section, the contingent basis of self-esteem can be measured using a validated questionnaire such as the Contingent Self-Esteem Scale—originally developed by Paradise and Kernis (1999; see also Kernis & Goldman, 2006; Kernis & Paradise, 2003). This involves 15 items that measure the degree to which a person’s sense of self-worth is dependent upon their own and others’ expectations. It is important to clarify that this scale measures self-esteem contingency and not self-esteem per se. What this means is that someone who with high self-esteem as measured by traditional methods such as the RSES could, in contrast, be measured as having low “contingent self-esteem” if their sense of self-worth is highly stable over time and independent of changing contexts (such as task failure) or others’ expectations. In other words, there is no direct relation between traditional measures of self-esteem and measures of contingent self-esteem.

The way in which self-esteem contingency might influence regret-based judgements in counterfactual reasoning has not been examined to date, yet seems to be an important avenue to explore. One relevant study is that reported by Greenier et al. (1999), which involved participants completing the RSES every 12 hours based on how they felt at that particular time, thereby permitting a measure of self-esteem stability. Next, participants were asked to write about a positive and a negative event that had occurred each day for a fortnight and also to provide a rating for how each event made them feel as well as a rating of each event’s negativity and importance. Greenier et al. found that negative events had a more negative influence on the feelings of those with unstable compared to stable self-esteem. Although Greenier et al. have identified this interesting phenomenon relating to the elevated level of reactivity to negative events for those with heightened contingent self-esteem, the authors remain moot as to a detailed explanation of such reactivity. They appear to see it simply as a product of self-esteem fragility, but this does rather beg the question of the theoretical mechanism underpinning this reactivity as well as its potential adaptive value.

Greenier et al.’s (1999) findings do, nevertheless, suggest that the relationship between unstable or contingent self-esteem and regret-focused counterfactual reasoning may likewise be consequential for people, such that those with an unstable or contingent self-esteem may respond more negatively when reasoning counterfactually about negative scenarios than those with more stable and less contingent self-esteem. This expectation formed a guiding framework for the study that we report subsequently, although as we discuss in the next section, our specific prediction in relation to the effect of contingent self-esteem on perceived regret intensity was also informed by the possibility that contingent self-esteem might show differential effects dependent on the *controllability* of the events associated with the regretted outcome (i.e., contingent self-esteem might modulate the way in which people reason about regretted incidents involving controllable vs. uncontrollable events).

## Counterfactual Reasoning and Event Controllability

A person’s perception of the controllability of the events that led to a negative outcome has been shown to be an important factor that is central to an understanding of counterfactual reasoning. In particular, studies have consistently demonstrated that people reason counterfactually about controllable events in a different manner to how they reason counterfactually about uncontrollable events. A classic study is that reported by Girotto, Legrenzi, and Rizzo (1991), who presented participants with a vignette about Mr. Bianchi, who had arrived home too late to save his wife, who was dying from a heart attack. There were three events that prevented Mr. Bianchi arriving home. Two of these events were uncontrollable: having an asthma attack, which meant he had to stop to take his inhaler, and accidentally breaking his spectacles, which meant that he had to return to his office to get a spare pair. One event, however, was controllable, that is, stopping at a bar to have a beer. When completing an “If only...” probe of the kind typically used to elicit counterfactual thinking, it was found that participants were more likely to undo the controllable event rather than any of the uncontrollable events. Girotto et al. argued that the increased tendency for reasoners to mutate controllable events arises because it is relatively easy for people to envisage the possibility where the controllable event simply did not occur (e.g., Mr. Bianchi did not stop for a beer), whereas it is more difficult to envisage the possibility where the uncontrollable events did not take place (e.g., Mr. Bianchi did not have an asthma attack).

Wilkinson, Ball, and Cooper (2010) examined how people reason about controllable and uncontrollable events using a think-aloud methodology. The think-aloud technique involves people speaking aloud their thoughts whilst they are tackling a given task. It is a technique that is assumed to provide reliable insights into the cognitive processes arising during task performance (see Ericsson & Simon, 1993). Think-aloud protocols have been shown to have superior validity compared to data that derive from either retrospective questioning or from people’s spontaneous remarks (e.g., Ericsson & Simon, 1993; Payne, 1994). The task that participants in Wilkinson et al.’s study had to complete was to state which of two protagonists would feel the most negative affect in a given scenario and to reason through how these two protagonists would be likely to experience the situation. Wilkinson et al.’s analyses of the resulting think-aloud protocols indicated that scenarios involving only controllable events evoked a greater level of highly engaged “mental simulation” compared to scenarios involving only uncontrollable events. Such simulation took the form of participants either pursuing extended reasoning about how they would feel in a given situation (e.g., Gordon, 1986) or imagining how another person would feel by placing themselves in their shoes and using the person’s likely belie*Fs* and desires to make inferences (e.g., Goldman, 2006). In contrast, the scenarios describing uncontrollable events evoked greater levels of “theory-based” reasoning (e.g., Carruthers, 1996) compared to the scenarios describing controllable events. Such theory-based reasoning was relatively rapid and immediate and involved participants drawing on general folk psychological theories about how people tend to behave and feel under certain conditions (e.g., people will be upset when they miss out on something).

Wilkinson et al.’s (2010) findings suggest that different cognitive processes are elicited when reasoning about controllable and uncontrollable events, with the former being more likely to trigger simulation-based counterfactual thinking that has the potential to engender deeper and more intense feelings of regret than that arising from theory-based reasoning in the case of uncontrollable events. Wilkinson et al. suggest that counterfactual reasoning arising through mental simulation might have adaptive value in helping people to be better prepared if similar situations arise again in the future (e.g., Epstude & Roese, 2011; Smallman & Roese, 2009; Wong, Haselhuhn, & Kray, 2012).

The aforementioned studies by Girotto et al. (1991) and by Wilkinson et al. (2010) involved the use of vignette-type regrets. A study examining *real-life* regrets and issues concerning event controllability was reported by Wrosch and Heckhausen (2002). They found that having a sense of control over a regretted situation—something that they termed “internal-control”—resulted in different psychological effects for different age groups. Whilst younger adults responded positively, reporting low levels of regret and low intrusive thoughts, older individuals responded with high levels of regret as well as high levels of intrusive thoughts. Such findings indicate that there are individual differences between groups regarding how people respond when they feel they have a sense of control (or not) over the event outcome.

## Predictions

In light of the aforementioned findings, such as those reported by Greenier et al. (1999), Girotto et al. (1991) and Wilkinson et al. (2010), we predicted that the regret intensity that people express for scenarios involving negative outcomes for controllable versus uncontrollable events would be modulated by the individual’s level of contingent self-esteem. More specifically, we predicted that: (1) people with high contingent self-esteem would reveal heightened regret intensity for regrets involving *controllable* events compared to people with low contingent self-esteem; and (2) people with high contingent self-esteem and low contingent self-esteem would show similar levels of regret intensity for regrets involving *uncontrollable* events.

These predictions are linked particularly closely to Greenier et al.’s (1999) observation that negative events have a more negative influence on the feelings of those with unstable (i.e., high contingent) compared to stable (i.e., low contingent) self-esteem. However, the prediction extends this observation to situations involving negative outcomes in the wake of controllable events of a type that could easily have been affected by the individual’s own actions. In relation to such regretted scenarios the assumption is that those with high contingent self-esteem, when reflecting on the regretted situation, would feel that their self-worth is particularly challenged by their own behaviors that they had control over, with such insecurities thence leading to heightened regret intensities relative to those with low contingent self-esteem. These latter individuals, whose self-esteem is robust and independent of expectations, are unlikely to feel that their self-worth is challenged even when reflecting on regretted situations where they had control over the events that led to a negative outcome. In the case of regrets involving uncontrollable events that led to negative outcomes, we assume that such situations would be viewed similarly by individuals with high versus low contingent self-esteem given that the events arose independent of any role that the individual played within the scenario. In the present research we addressed head on this novel prediction concerning the interaction between event controllability for regretted outcomes and contingent self-esteem.

## Method

### Participants

An opportunity sample of 109 participants was recruited for the study from a UK University campus. There were 52 males, 50 females and 7 gave no information regarding gender. Participants were aged between 18 and 61 years (*M*_age_ = 22.7 years, *SD*_age_ = 5.5 years). Because of technical errors with our study booklets as well as missing regret data we reduced our sample to 85 participants (i.e., 42 males, 39 females and 4 who gave no information regarding gender; age range = 18 and 44 years; *M*_age_ = 23.0 years; *SD*_age_ = 5.4 years).

### Design

A mixed within-between participants design was adopted, with a within-participant factor of regret condition with two levels, that is, controllable events versus uncontrollable events, and a between-participants factor of contingent self-esteem with two levels, that is, low contingent self-esteem versus high contingent self-esteem. The levels of the self-esteem factor reflected a median split of the self-esteem data (see below for further discussion).

### Materials and Procedure

Participants were tested individually or in small groups and were asked to complete a questionnaire booklet containing the Paradise and Kernis (1999) Contingent Self-Esteem Scale (see also Kernis & Goldman, 2006; Kernis & Paradise, 2003; Zeigler-Hill, Besser, & King, 2011). Participants are given 15 statements and have to rate how much each statement is like them on a 5-point scale (1 = *not at all like me*, 3 = *neutral*, and 5 = *very much like me*). These statements either correspond to perceived expectations of others (e.g., “My overall feelings about myself are heavily influenced by how much other people like and accept me”) or to self-expectations (e.g., “Even in the face of failure, my feelings of self-worth remain unaffected”). After completing the questionnaire participants’ scores were aggregated (with some items reverse coded) so as to give a total score of self-esteem contingency, with a higher score reflecting more contingent self-esteem that is unstable, insecure, and fragile in nature.

Participants were asked to generate two personal regrets from their lives, one that involved events that were completely within their control and the other that involved events that were completely outside of their control. The order of regret generation (controllable or uncontrollable) was counterbalanced across participants, as was whether they generated these regrets before or after completing the Contingent Self-Esteem Scale. An example of a controllable regret might be leaving an assignment to the last minute due to engaging in an overabundance of social activities, whereas an example of an uncontrollable regret might be missing an assignment deadline having had a loved one pass away through a sudden illness. Participants were asked to write about each regret in as much detail as possible and rate their regret intensity on a 7-point scale (1 = *little regret*; 7 = *a great deal of regret*). They were also requested to rate their sense of control over the regret, again using a 7-point scale (1 = *fully out of my control*; 7 = *fully in my control*). Participants responded to these questions as part of a larger study examining the nature of people’s reasoning about regrets. After completing the study participants were debriefed. The study was approved by the University Ethics Committee.

## Results

For all of the statistical analyses reported below the alpha level was set at .05.

### Contingent Self-Esteem Scale: Reliability Checks and Group Membership Criteria

The internal consistency of the Contingent Self-Esteem Scale for our sample was acceptable (Cronbach’s alpha = .80) and in line with what has been found by other researchers (e.g., Zeigler-Hill, Besser et al., 2011). Most studies examining self-esteem and counterfactual thinking have adopted a median split to separate participants into different self-esteem groups (see Feeney et al., 2005; McDaniel & Pettijohn, 2013). For consistency with the extant literature we adopted the same approach, with our median split resulting in a low contingent self-esteem group (*N* = 51, *M* = 46.20, *SD* = 5.07, range = 31 to 52) and a high contingent self-esteem group (*N* = 34, *M* = 58.71, *SD* = 3.98, range = 53 to 70).

### Controllable Versus Uncontrollable Regrets: Manipulation Checks

Our main experimental manipulation was regret controllability, with participants being requested to generate two regrets, one involving controllable events and the other involving uncontrollable events, which they then rated for controllability and regret intensity. Before progressing to an analysis of the regret intensity data we first conducted a manipulation check to determine that participants’ self-generated regrets polarized effectively on the 7-point controllability scale that they used to rate them.

To undertake this manipulation check we conducted a 2 × 2 mixed-design ANOVA on the controllability ratings (see Table 1 for mean data), where the between-participants factor was contingent self-esteem group (high vs. low contingent self-esteem) and where the within-participant factor was regret type (i.e., controllable vs. uncontrollable events). This analysis revealed a significant main effect of regret type, with controllable regrets being rated as being associated with a greater sense of control over events than uncontrollable regrets, *F*(1, 83) = 133.05, *MSE* = 2.29, *p* < .001, η_p_^2^ = 0.62. This finding therefore confirms the effectiveness of the regret type manipulation. Importantly, there was no effect of self-esteem group on controllability ratings, *F* < 1, and neither was there a significant interaction between regret type and self-esteem group, *F*(1,83) = 1.60, *MSE* = 2.29, *p* = .21, η_p_^2^ = 0.02.

**Table 1.** Mean Ratings of Feelings of Control for Different Regret Types as a Function of Level of Contingent Self-Esteem

Regret Type			
Self-esteem group	Controllable	Uncontrollable	Mean
High contingent self-esteem	5.37 (1.41)	2.94 (1.98)	4.16
Low contingent self-esteem	5.68 (1.47)	2.65 (1.81)	4.17
Mean	5.53	2.80	

*Note.* *SD* in parenthesis.

### Event Controllability, Regret Intensity, and Contingent Self-Esteem

Our key experimental predictions were that people with high contingent self-esteem would show heightened regret intensity for regrets involving *controllable* events compared to people with low contingent self-esteem, whilst people with high contingent self-esteem and low contingent self-esteem would show similar levels of regret intensity for regrets involving *uncontrollable* events. Our findings appear to support these predictions. As can be seen from Table 2, for the controllable regret type the high contingent self-esteem group reported higher regret intensity than the low contingent self-esteem group, whereas for the uncontrollable regret type there are little differences in rated regret intensities across the two self-esteem groups^1^.

**Table 2.** Mean Ratings of Regret Intensities for Different Regret Types as a Function of Level of Contingent Self-Esteem

Regret Type			
Self-esteem group	Controllable	Uncontrollable	Mean
High contingent self-esteem	5.47 (1.50)	5.24 (1.56)	5.36
Low contingent self-esteem	4.76 (1.58)	5.34 (1.56)	5.05
Mean	5.12	5.29	

*Note.* *SD* in parenthesis.

A 2 × 2 mixed-design ANOVA was conducted on regret intensity ratings, where the between-participants factor was contingent self-esteem group (high vs. low contingent self-esteem) and where the within-participant factor was regret type (controllable vs. uncontrollable events). This analysis revealed that there was neither a main effect of self-esteem group, *F*(1, 83) = 1.16, *MSE* = 3.16, *p* = .29, η_p_^2^ = 0.01, nor a main effect of regret type, *F* < 1. In line with predictions, however, there was a significant interaction between regret type and self-esteem group, *F*(1, 83) = 4.03, *MSE* = 1.68, *p* < .05, η_p_^2^ = 0.05.

To clarify the effects underlying this significant interaction in relation to our predictions we conducted separate simple main effects analyses for the controllable and for the uncontrollable regrets. For controllable regrets we found a significant simple main effect of contingent self-esteem group, whereby the regret intensity of controllable regrets was rated higher by individuals with high contingent self-esteem than individuals with low contingent self-esteem, *F*(1, 83) = 5.05, *MSE* = 2.36, *p* < .05, η_p_^2^ = 0.05. For uncontrollable regrets no significant simple main effect was in evidence, *F* < 1. These simple main effects analyses conform to the predicted modulating effect of contingent self-esteem on controllable versus uncontrollable regret types.

We corroborated these latter effects using linear regression analyses in which we treated contingent self-esteem as a continuous variable rather than as a dichotomous variable. The first model that we examined was for controllable regret types, with the continuous predictor being contingent self-esteem and the dependent variable being regret intensity. This model was found to be reliable, *R* = .24, adjusted *R*^2^ = .05, *F*(1, 84) = 5.20, *p* < .025. The second model that we examined was for uncontrollable regret types, with the continuous predictor again being contingent self-esteem and the dependent variable being regret intensity. This model was not reliable, *R* = .11, adjusted *R*^2^ = .001, *F*(1, 84) = 1.07, *p* = .34.

### Regret Intensity Ratings and Order Effects

We conducted a simple check to determine whether question ordering had any impact on regret intensity ratings, bearing in mind that the order of regret type was counterbalanced in our experimental design. We conducted a 2 × 2 mixed-design ANOVA on regret intensity ratings, where the within-participant factor was regret type (controllable vs. uncontrollable) and where the between-participants factor was order of regret type (controllable first vs. uncontrollable first). This ANOVA revealed that there was no influence of regret type order, neither as a main effect nor in interaction with regret type, both *Fs* < 1.

We finally ascertained whether completing the self-esteem questionnaire before or after generating and rating the two regret types had any impact on regret intensity ratings. To do this we conducted a 2 × 2 mixed-design ANOVA on regret intensity ratings, where the within-participant factor was regret type (controllable vs. uncontrollable) and where the between-participants factor was order of responding (regrets first vs. self-esteem questionnaire first). Again, this ANOVA revealed that there was no influence of response order, either as a main effect or in interaction with regret type, both *Fs* < 1.

## General Discussion

Existing research on the association between self-esteem and counterfactual reasoning has used either the Rosenberg Self-Esteem Scale (Rosenberg, 1965) to assess the impact of self-esteem on the recall of regretted life events (Feeney et al., 2005) or the Texas Social Behavior Inventory (Helmreich & Stapp, 1974), to assess the aspects of regretted events that are mutated to undo a negative outcome (Roese & Olson, 1993). This research has failed to recognise that self-esteem is a dynamic construct that varies in its stability over time and contexts (Kernis, 2005; Kernis & Waschull, 1995; Zeigler-Hill, Besser et al., 2011; Zeigler-Hill, Fulton et al., 2011). For some, self-esteem is highly contingent upon personal expectations that need to be lived up to or expectations that others are believed to have of them (Deci & Ryan, 1995; Kernis, 2005). To date no studies appear to have examined the relation between contingent self-esteem and regretted events associated with longer-term counterfactual thinking.

In the present research we set out to bridge this gap in our understanding by examining the influence of contingent self-esteem (e.g., Kernis, 2005; Kernis & Waschull, 1995) on regret intensity for controllable versus uncontrollable regret types. The former involve prior events associated with a negative outcome that were under one’s control (e.g., acts of commission or omission), whereas the latter involve prior events associated with a negative outcome that were outside of one’s control (e.g., accidents or misfortunes). Previous studies suggest that people show an increased tendency to mutate controllable events rather than uncontrollable ones, presumably because it is more difficult to envisage the possibility of an uncontrollable event not having happened (e.g., Girotto et al., 1991).

Notwithstanding Girotto et al.’s (1991) findings, the question of whether contingent self-esteem has a modulating influence on judgments relating to controllable and uncontrollable regret types has remained unanswered until the present study. In formulating our research we derived the prediction that for controllable regret types it would be individuals with high contingent self-esteem who would report greater regret intensity relative to individuals with low contingent self-esteem, whereas for uncontrollable regret types there would be no difference between groups in regret intensity ratings. This prediction was informed by findings reported by Greenier et al. (1999) indicating that individuals with unstable or contingent self-esteem respond more negatively when reasoning counterfactually about negative-outcome scenarios than those with more stable and less contingent self-esteem. Such a finding may arise as a consequence of participants feeling responsible for the outcome, since such events challenge their perceived sense of self-worth (e.g., when receiving a poor mark for an assignment they begin to doubt themselves rather than viewing the mark as just a one-off occurrence that can be attributed, say, to a strict marker). The results of our study supported the predicted modulation of regret type by contingent self-esteem in relation to regret intensity responses, that is: (1) for controllable regret types those individuals with high contingent self-esteem reported greater regret intensity relative to individuals with low contingent self-esteem; and (2) for uncontrollable regret types there was no difference in regret intensity ratings for those with high versus low contingent self-esteem.

In interpreting this predicted modulation effect arising from individual differences in contingent self-esteem we propose that individuals with high contingent self-esteem respond to regretted incidents that they have control over by invoking thoughts to the effect that they have failed to live up to their own or others’ expectations. Such thoughts will promote deeper feelings of regret than arise for individuals with low contingent self-esteem, whose feelings of self-worth are more stable, less expectation-driven, and less in need of validation. Our results align with the findings of Kernis and Paradise (2003), who showed that more negative affect (in the form of *anger*) occurred for those with a high contingent self-esteem in the wake of negative feedback in comparison to those with low contingent self-esteem.

Our findings, together with those of Kernis and Paradise (2003) and Greenier et al. (1999), suggest that people with high contingent self-esteem respond in a more negative, hostile, or intense manner than those with low contingent self-esteem in the wake of negative outcomes. We suggest that this mode of responding may have at its core the application of adaptive mechanisms that are directed toward future self-achievement in relation to personal expectations. For example, in the case of having intense regret for controllable events that led to negative outcomes an individual with high contingent self-esteem can engage in a process of counterfactual reasoning about how things could have turned out differently, which can thereby elicit planning for the future (Epstude & Roese, 2011). By engaging in such planning, high contingent self-esteem individuals can feel that the expectations that determine their self-worth can be lived up to at a future time.

This account also explains why we found no evidence for contingent self-esteem having an influence on regret intensity for regretted outcomes associated with uncontrollable events. In the wake of a negative outcome for events outside of one’s control one’s sense of self-worth remains unchallenged since one could not have influenced the outcome anyway. Although we did not obtain any direct measure of how participants were reasoning about regretted situations, the fact that imagined events can be mutated more easily for controllable than uncontrollable regrets (Girotto et al., 1991; Wilkinson et al., 2010) provides a mechanism whereby individuals with high contingent self-esteem can ascertain how they could have done things differently so as to gain insight into meeting expectations for effective behaviour in the future.

Our data also suggest that individuals with low contingent self-esteem may be actively engaged in ego-protection and self-enhancement processes in dealing with regretted scenarios involving controllable events. This possibility is evidenced by the fact that their regret intensity ratings for these scenarios were substantially lower than those for scenarios involving uncontrollable events, which is a curious result unless one assumes that low contingent self-esteem individuals are down-playing their causal role in situations involving a regretted outcome and also disengaging from the plan-oriented thinking that could promote improved outcomes in similar, future scenarios. Such ego-protection resonates with the proposals of Feeney et al. (2005), who present evidence that people with high self-esteem self-enhance by distancing themselves from taking responsibility for bad outcomes (cf. Brown & Mankowski, 1993).

Although we have presented a positive account of the role that high contingent self-esteem can play for future planning and expectation attainment we recognise there is a downside to having highly unstable or contingent self-esteem in that the individual will constantly need to seek validation from either themselves or from others for their behaviours, which is not viewed as psychologically healthy (e.g., Borton, Crimmins, Ashby, & Ruddiman, 2012; Crocker, Karpinski, Quinn, & Chase, 2003; Crocker & Knight, 2005; Kernis, Paradise, Whitaker, Wheatman & Goldman, 2000; Park & Crocker, 2005). Furthermore, if one’s contingent self-esteem is too high then this may lead one to become very negatively affected and potentially de-motivated to try to do better in the future. At the same time it is also not psychologically healthy to have very *low* contingent self-esteem, since event outcomes will not challenge one’s sense of self-worth so as to motivate one to do things better next time and avoid attributing failures to others.

More research is needed to explore the links between different degrees of self-esteem contingency and these motivational and expectation-attainment aspects of behaviour linked to future action-planning and it is noteworthy that researchers have recently started to examine the relation between self-esteem contingency and self-esteem level. For example, Zeigler-Hill, Besser et al. (2011) adopted both the RSES and the Contingent Self-Esteem Scale to examine how people predict they will feel faced with situations of interpersonal rejection and failure. They found that those individuals with a high contingent self-esteem predicted that they would feel greater negative affect in the wake of these outcomes relative to individuals with a low contingent self-esteem. It would be useful to examine the relation between self-esteem and self-esteem contingency in future research involving controllable and uncontrollable event outcomes. Another important point to note concerns the link between self-esteem and depression. A recent longitudinal analysis that examined the relation between depressive symptomology, contingent self-esteem, and self-esteem level found that the latter was a unique predictor of depressive symptomology, whilst contingent self-esteem did not predict depressive symptoms when taking self-esteem level into account (Wouters et al., 2013). Such findings reinforce the importance of Zeigler-Hill, Besser et al.’s (2011) study examining self-esteem level and contingency together.

We finally note a potential confound in our study that may limit the interpretation of our findings, which relates to the fact that participants were requested to *generate* regretted events prior to rating these for intensity and event controllability^2^. Using this methodology it is possible that high and low contingent self-esteem individuals differ in the regret scenarios that they can think of, with high contingent self-esteem participants generating controllable regret types that are “objectively” more intensely regrettable than those generated by low contingent self-esteem participants. This is clearly an issue that needs to be addressed in follow-on research. We suggest that a good way forward would be to present individuals with tightly controlled regret-oriented scenarios experienced by another protagonist and then ask for intensity and controllability ratings. Such highly controlled materials would clarify whether high contingent self-esteem continues to be associated with elevated regret intensity, thereby corroborating and generalising the effects observed in the present study.

To conclude, this paper has demonstrated that people with high contingent self-esteem report greater regret intensity than people with low contingent self-esteem for controllable but not for uncontrollable event outcomes associated with regrettable incidents. In other words, a person’s perceived regret for controllable versus uncontrollable regret types is modulated by their contingent self-esteem. We suggest that this novel finding indicates that contingent self-esteem may be functional in terms of mobilizing the application of counterfactual reasoning and planning mechanisms that can enable personal expectations to be achieved in the future (cf. Epstude & Roese, 2011). We further propose that research on counterfactual reasoning needs to move away from the issue of whether one’s self-esteem is low or high, as in most previous studies (e.g., Feeney et al., 2005), and instead focus more on the effects of self-esteem contingency and stability. We hope that our study goes some way toward expanding this important research avenue.

## Footnotes

1 The regret intensity data presented in Table 2 indicate that there was a tendency (albeit non-reliable) for participants to have more intense levels of regret for situations involving uncontrollable events compared to situations involving controllable events. These findings align well with those reported by Wrosch and Heckhausen (2002), who showed that younger adults of similar mean age to our participants likewise showed heightened regret intensity for “low control” relative to “high control” regrets.

2 We are very grateful to the Action Editor who handled our manuscript for alerting us to this potential confound.
